# Naloxone and the Inner City Youth Experience (NICYE): a community-based participatory research study examining young people’s perceptions of the BC take home naloxone program

**DOI:** 10.1186/s12954-017-0160-3

**Published:** 2017-06-07

**Authors:** Keren Mitchell, S. Elise Durante, Katrina Pellatt, Chris G. Richardson, Steve Mathias, Jane A. Buxton

**Affiliations:** 1 Inner City Youth Program, 1260 Granville Street, Vancouver, BC V6Z 1M4 Canada; 2 School of Nursing, University of British Columbia, Vancouver, Canada; 3 School of Population and Public Health, University of British Columbia, 2206 East Mall, Vancouver, BC V6T 1Z3 Canada; 4 BC Centre for Disease Control, 655 West 12th Ave, Vancouver, BC V5Z 4R4 Canada

**Keywords:** Naloxone, Narcan, Youth, Young adult, Take home naloxone, Harm reduction, Opioids, Participatory research, Mental health

## Abstract

**Background:**

Take home naloxone (THN) programs reduce mortality by training bystanders to respond to opioid overdoses. Clinical observation by the health care team at the Inner City Youth (ICY) program indicated that young adults appeared to enthusiastically participate in the THN program and developed improved relationships with staff after THN training. However, we found a dearth of literature exploring the experiences of young adults with THN programs. This study set out to address this gap and identify suggestions from the young adults for program improvement. The primary research question was “How do street-involved young people experience the THN Program in Vancouver, BC?”

**Methods:**

The study was undertaken at the ICY Program. Two peer researchers with lived experience of THN were recruited from ICY and were involved in all phases of the study. The peer researchers and a graduate student facilitated two focus groups and five individual interviews with ICY program participants using a semi-structured interview guide. Audio recordings were transcribed verbatim. The cut-up-and-put-in-folders approach was used to identify emerging themes.

**Results:**

The themes that emerged were perceptions of risk, altruism, strengthening relationship with staff, access to training, empowerment, and confidence in ability to respond, and suggestions for youth-friendly training. These themes were then situated within the framework of the health belief model to provide additional context. Participants viewed themselves as vulnerable to overdose and spoke of the importance of expanding access to THN training. Following training, participants reported an increase in internal locus of control, an improved sense of safety among the community of people who use drugs, improved self-esteem, and strengthened relationships with ICY staff. Overall, participants found THN training engaging, which appeared to enhance participation in other ICY programming.

**Conclusions:**

Young people perceived THN training as a positive experience that improved relationships with staff. Participant recommendations for quality improvement were implemented within the provincial program.

**Electronic supplementary material:**

The online version of this article (doi:10.1186/s12954-017-0160-3) contains supplementary material, which is available to authorized users.

## Background

Providence Health Care’s Inner City Youth (ICY) Program is a mental health and addiction team which support young people aged 16 to 24 years at the time of intake who are homeless or precariously housed in Vancouver, British Columbia (BC). To qualify for the Intensive Case Management (ICM) stream, young people present with moderate to severe mental illness and/or substance use disorders (as defined by the American Psychiatric Association *Diagnostic and Statistical Manual of Mental Disorders* (5th ed.) For many, opioids are their drug of choice. Young people in the ICM stream are eligible for a range of services, including psychiatric care, primary care, psychosocial rehabilitation, and supportive housing.

Opioids are a class of drugs that relieve pain and are central nervous system (CNS) depressants. Heroin, fentanyl, methadone, and hydromorphone are among the more commonly used opioids among ICY clients. In part due to opioid’s action as a CNS depressant, (which slows and even stops breathing) there is a relatively high prevalence of witnessing or experiencing overdose among people who use intravenous, non-prescription opioids [1, 2]. Specific to ICY, many ICY clients are involved in behaviors that increase their risk of overdose, such as injecting drugs, mixing other substances with opioids, and using after periods of abstinence. Naloxone is a pure opioid antagonist that when administered during an opioid overdose will reverse the effects of CNS depression.

Take home naloxone (THN) programs involve providing naloxone and teaching people how to prevent, recognize, and respond to an opioid overdose, including seeking professional help, rescue breathing, and how to correctly administer naloxone [3]. Most overdoses occur in the presence of other people, including peers, family members, and others [4]. Literature shows that people who use opioids and their support people can be effectively trained to identify and respond to an overdose with THN [5]. Knowledge and comfort with overdose identification and response improves after THN training [6]. The risk involved in administering naloxone is minimal [7]. There are THN programs in Canada, the USA, Europe, Asia, and Australia [4, 8].

ICY staff noticed that young adults appeared eager to participate in the THN program, and staff-client relationships seemed to be enriched after participating in the THN training. To date, studies on THN programs have predominantly focused on people with longer-term opioid use, and there is a lack of literature on the impact of THN training on young adults. This study set out to address this knowledge gap by investigating the experience of THN training among young adults at ICY who had a history opioid use. The authors also explored how participating in the THN program affected engagement in other aspects of mental health and addictions programming and collected suggestions on how to improve the THN program.

The BC Centre of Disease Control (BCCDC) implemented the BC THN program in August 2012. The BCCDC develops the training materials and provides registered sites with free THN kits and training supplies for people who are personally at risk of overdose. Sites complete training and kit distribution records, and assist clients in filling out naloxone administration forms after a person has responded to an overdose event. The sites send all records to the BCCDC to collate. The program was found to be “easy to implement and empowering for clients” [9]. The ICY program became a THN training site on October 1, 2013. As of January 2017, 94 youth and 195 support people had been trained in THN by ICY, and there were 55 THN kits reported to be used by ICY participants to respond to overdose.

A public health emergency was declared in British Columbia (BC) on April 14, 2016 by the Provincial Health Officer, due to a substantial increase in opioid overdose-related injury and death across the province [10]. Recent reports from the BC Coroners Service identify 366 illicit drug overdose fatalities in BC in 2014, 514 in 2015, and more than 900 deaths in 2016 [11]. In addition, the proportion of overdose deaths known to be linked to fentanyl has increased from 4% in 2012 to 62% in 2016 [12]. As a result, a federal policy change occurred to improve access to naloxone. When this study began, naloxone was available by prescription only, and people at personal risk of overdose could be prescribed a kit only after completing THN training. Health Canada removed naloxone from the Schedule 1 prescription drug list in March 2016 [13]; naloxone is now available over the counter in pharmacies in BC. The BC THN program makes kits available at 492 sites at no cost to people who use opioids, and kits are now available to family and friends. In 2016 alone, more than 22,000 kits were dispensed and 4200 kits were reported used to reverse an overdose [14].

THN programs effectively train people to respond to an overdose by using naloxone. Young adult participants articulated the need for easy access to THN, and this finding has been consistent across current press coverage on this topic [15]. This study aimed to address a gap in the literature and provide insights about how young adults perceive and interpret the effect that THN programs have upon them and their behaviors, and to explore how providing THN programming may influence their relationship with their health-care team. These findings will inform changes in THN and other programs at ICY to increase engagement and improve outcomes.

## Methods

Principles of community-based participatory research (CBPR) informed the study design. CBPR supports the inclusion of the community members who are from the group that is being researched. Two peer researchers, with lived experience of THN, were recruited from young adults attending ICY. The peer researchers received training on research methods alongside the co-investigators. The “ladder of participation” is a model that measures peer involvement in CBPR from non-participatory and tokenized levels on the lower rungs, to active and equal involvement with researchers on the higher rungs. The research team strove to work as equal partners with youth researchers to ensure that peer involvement was on the higher rungs of the ladder. Therefore, shared decision-making was employed in all areas of the study, including design, interviewing subjects, data interpretation, and information dissemination. Peer participation in research increases relevance, and assists in more culturally appropriate data collection, analysis, and dissemination [16]. We used a phenomenological approach to bring the experiences and interpretations of the situation (opioid overdose and THN) from the individuals’ or actors’ (young adults) own perspectives [17]. Ethical approval was obtained from the Behavioral Research Ethics Board at the University of British Columbia.

### Participants and sampling

At the time of the study development in December 2014, 103 individuals were enrolled in the Intensive Case Management stream of ICY; 34 (33%) of these were identified by their case manager as using opioids and 27 (79%) of these met DSM-5 criteria for moderate to severe opioid use disorder [18]. Diagnoses of substance use disorders were made by an ICY physician or by a nurse practitioner.

Recruitment occurred through posters, word of mouth, and announcements in ICY group sessions. Purposive sampling methods ensured participants were from the Intensive Case Management stream and met eligibility criteria of age 19–25 years, self-identified as having used opioids, had received THN training by an ICY team member, and included youth who identified as male and female. ICY follows youth who are 24 years old at the time of intake; however, young people may still be enrolled in the program at age 25 while planning transition to an adult service. Youth under 19 years were excluded from this study due to ethical concerns. Members of the research team who work as clinicians at ICY were not directly involved in participant recruitment or data collection. It was explicit that participation was voluntary and would not affect a participant’s access to clinical services. Interested young adults contacted a non-clinical team member to enroll and were provided with written informed consent prior to their participation. Participants completed a brief demographic questionnaire regarding age, gender, and recent substances used.

### Data collection

Data were collected through focus groups and individual interviews conducted by peer researchers and a Masters of Public Health (MPH) student in July and August 2015. Interviews and focus groups took place in ICY offices and at the Granville Youth Health Centre, where participation was confidential and participants were familiar with the surroundings. Peer and staff researchers developed a semi-structured interview questionnaire. Participants were advised that mental health support was available if needed during or after the interview or focus group. Participants received a small honorarium of $20 CDN to compensate for their time and for sharing their experience and snacks were provided. (focus group and individual interview questionnaires are included as Additional files 1 and 2).

### Data analysis

Focus groups and interviews were audio recorded and transcribed verbatim. All identifying information were removed; researchers in a clinical role with ICY clients were not permitted to know the identity of participants. Team members read transcripts and analyzed data manually as a group. The team adapted the “cut-up-and-put-in-folders approach” by which “meaning units” were identified and cut out with source information to provide context. [19] The team members took turns to read each quote aloud and the team discussed and coded each quote. The team identified emerging themes and placed the cut-out text in the corresponding “theme” envelope. As the iterative process continued, the team revisited the themes and decisions to reorganize (divide, combine, or rename) themes were determined by consensus. The process continued until team members came to agreement of the key themes, quotes, and meaning of data. This approach was selected rather than using qualitative data analysis software so that the research team could discuss each transcript in detail, which was possible given the relatively small data set. This resulted in a nuanced understanding of participant responses. Additionally, the use of analysis software is complex, and may have limited the ability of team members to participate equally. A peer researcher was involved at all times during data analysis to increase relevance, which is aligned with the CBPR approach.

## Results

In total, 11 young adults participated in 2 small focus groups of 2 and 4 participants, and 5 individual interviews. Focus groups were small because not all enrolled participants arrived for the scheduled sessions. Young people who missed the focus groups were offered the option of an individual interview. Seven participants identified as male, four as female, and none identified as transgender; nine participants reported using opioids in the preceding week. Opioids reported used included heroin, hydromorphone, morphine, and methadone. Eight participants also identified using methamphetamine in the preceding week. Other substances participants reported having used in the previous week included cannabis (*n* = 5), cocaine (*n* = 2), alcohol (*n* = 1), and benzodiazepines (*n* = 1).

The major themes that emerged were perceptions of risk, motivations for participating in training, altruism, strengthening relationships, and the importance of accessible naloxone, empowerment, and suggestions for youth-friendly improvements to THN education and service delivery.

We considered the major themes identified in the context of various health behavior models and theories; the health belief model was determined to be the most appropriate framework to illuminate the findings. The health belief model focuses on the individual’s attitudes and beliefs; it originally had four constructs: perceived susceptibility, perceived severity, perceived benefits, and perceived barriers. Two additional concepts have been added cues to action (strategies to activate “readiness”) and self-efficacy (confidence in one’s ability to take action) [20]. Empowerment can be considered a consequence of achieving self-efficacy and gaining mastery over one’s own life [21].

Table 1 shows how the identified themes aligned with the core components of the health belief model. Many of the comments identified by participants as quality improvement were suggestions to reduce barriers and improve self-efficacy.

**Table 1:** Alignment of the identified themes with the health belief model

Health belief model	Themes identified
1. Perceived threat (perceived susceptibility and perceived severity)	Perceptions of risk
2. Perceived benefits	Motivation for participating in training: altruism Improved relationships with staff
3. Perceived barriers	Access to training
4. Cues to action (strategies to activate “readiness”)	Training and awareness
5. Self-efficacy (confidence in one’s ability to take action)	Empowerment and improved self-esteem; confidence in ability to respond: Youth relevant video ‘Dummy’ for training Offer repeat training/refreshers

1. Perceived threat:
  Perceptions of risk
  Young adult’s understanding of overdose risk developed over time, and was influenced by close encounters with overdose. Participants said that personally experiencing or witnessing an overdose made them acutely aware of their vulnerability, which was a source of concern as they recognized themselves at personal risk of overdose.
  “-- it really kind of wakes you up and makes you realize that it’s real life and you’re kind of playing with fire sometimes.” Participant 3
  “Yeah, it’s just -- you can never really prepare yourself. It’s, I mean, you think going into it that you’re invincible and that you’re going to be able to notice when you’re going to start slipping into an overdose and that things are going to be okay for you, but you’re the same as everyone else. You can fall victim to the same casualty.” Participant 8
2. Perceived benefits:
  Altruism
  Throughout the interviews and focus groups, participants spoke a sense of altruism towards their peers, family, and community as reasons to carry naloxone and receive training in overdose response. Participants identified the ability to save other lives as a powerful motivator to participate in the training. They felt they had a responsibility to take care of other people who use substances, and they perceived this responsibility in a positive way.
  “You can save a person’s life by using this stuff because I had first-hand experience and I know that the stuff works.” *Pa*rticipant 2
  “Because a family member close to me died from an overdose. And I kind of think of it as, like, in honor of her, you know.” Participant 9
  THN training appeared to increase the sense of safety for the participants. They felt more aware of what to do in the event of an overdose, and they believed that naloxone would be effective.
  “I felt like I could actually do something to stop their overdose.” Participant 5
  Relationships with staff
  Participants linked the training in overdose prevention to being genuinely cared for, valued, and respected by staff. They reported feeling that they were treated with compassion and not judged, which contrasted with their expectations to experience negative reactions linked to their drug use.
  “I think it, like, really [inaudible] reinforced and, like, people [ICY staff] are genuinely caring about us. And-you guys are a bunch of junkies, you don’t fucking need to, like-were actually, like, really gave a shit and were, like, considerate and polite.” Participant 6
  “I like that they’re [ICY staff] aware that not all drug users are, like, scum or anything. They treat us well. And they’re preparing us for like possible death and I like that it’s, like, harm reduction in general.” Participant 7
  “Well, we’re not looked down upon just because we use. And, like, stigma is not changing the relationship between a user and a non-user.” Participant 9
  Participants spoke about recognizing that ICY staff wanted to speak with them candidly about their substance use and harm reduction. Young adults expressed that the THN program provided them with a valuable skill, and improved their relationships with ICY staff. After THN training, they felt they could speak openly about issues that they previously were not comfortable discussing. Young people recognized that ICY staff were genuinely concerned and wanted them to be safe, which improved trust and helped them feel less self-conscious discussing substance use.
  “They [ICY staff]-- like, I guess they knew I was at a point where I was doing some pretty dangerous things. So they wanted -- didn’t want to see anything bad happen to me, so they really pushed for me to do the Naloxone training.” Participant 4
  “Like, it almost feels like a weight has been lifted or kind of like - I don’t know how to describe it, but when a layer has been, like, taken or, like, there’s an invisible wall that was broken. And we can now talk about something a bit more casually, even though it’s serious.” Participant 9
  Empowerment and improved self-esteem were reported by participants as perceived benefits of the program, but were also identified as improving confidence in one’s ability to take action and are therefore addressed in “self-efficacy” section.
3. Perceived barriers and cues to action:
  Access and awareness of training
  Participants repeatedly spoke about the need to have increased access to naloxone and THN training. They suggested that ICY offers training universally to all clients, and that THN kits be available in public places. An example of this is the suggestion to keep naloxone in ‘hotels.’ (In this context ‘hotel’ refers to single room occupancies (SRO), which are low income housing units with shared bathrooms and cooking areas. SRO hotels are often low-barrier and first-stage housing for people who have been homeless.)
  “They should actually have, like, a fire extinguisher, a case, in every hotel [SRO] that people use drugs with Naloxone.” Participant 8
  “…the training should be a part of ICY. It should be mandatory for people who live in a hotel. You have a choice not to do it, but I totally recommend taking the training because I was totally oblivious to Narcan. I didn’t know what it was. I thought it was just a stimulant.” Participant 2
4. Self-efficacy:
  Empowerment and improved self-esteem
  Participants were aware of the risk, but felt powerless to respond to overdose prior to the training. THN training increased their sense of agency.
  “Well, because you, like, before when you don’t have naloxone training, you don’t have a naloxone kit, when an overdose comes around and what can you really -- you can, like [inaudible] said, you can give a person CRP (sic) and pray and hope for the best. Whereas with naloxone you’re kind of given the power to try to do something about it.” Participant 8
  Participants who had used naloxone previously talked about a sense of accomplishment and increased self-esteem. They felt empowered to be skilled in this intervention, and they recognized that this skill was valued and respected by peers, workers, family members, and other people in the community.
  “So the ambulance came and they basically took over. They said ‘Great job. You could have saved his life, you did awesome. We’re going to take it from here.” Participant 3
  “It was very satisfying. Very, like, accomplishing. I was glad I had helped someone.” Participant 1
  Confidence in ability to respond
  Mixed feelings were expressed by young adult participants about their comfort and confidence in their capability to respond to overdose following training. While some young people expressed confidence in their ability to respond to overdose, others stated that they felt somewhat underprepared. Interestingly, one of the participants who described feeling underprepared also reported having successfully reversed an overdose following the training.
  “Yeah, well, I guess right after I first got it, I’ve never actually done it before so—you know, watching it on a video and getting a little piece of paper saying I can do it and having the little kit on me and actually being able to do it or two different things. Yeah, the first time I did it I was a bit scared. But, yeah, there was someone there try—you know, calming me down and, you know, making sure I was, like, okay. And I was on the phone with the paramedics or whatever.” Participant 1
  Participants provided concrete suggestions for ways to improve the training and make it more youth-friendly, such as updating the training video, using training dummies, and making refresher training available. Young adults felt these changes would make the THN training more practical and would increase their confidence in their ability to resuscitate someone from an overdose.
  “They could have, like, you know, brought in a dummy or something and shown … what positions to put the person’s body or what to do to help them breathe or whatever.” Participant 1
  “It might be good to do a refresher every now and then.” Participant 3
  “The video was a bit dated.” Participant 8

## Discussion

Working within a framework of harm reduction, our primary research question was prompted by clinical observations that young adults appeared to positively engage with THN training. The community-based participatory research (CBPR) design intended to promote maximal involvement of service users, a pillar of harm reduction [22], in all aspects of research, quality improvement, and knowledge dissemination. CBPR promotes engagement among street-involved young people [16] and supports user-driven quality improvement in vulnerable populations [23]. The health belief model organized and grounded our interpretations of how THN training affects individual’s attitudes and beliefs.

Themes that emerged from the ICY study included perception of risk, motivation to take the training, empowerment, strengthening relationships with staff, access and importance of THN, and youth-friendly improvements. THN training increased a sense of safety at a time when the risk of overdose was high. Receiving THN training also fosters a sense of agency and increased self-esteem, with a newly acquired skill recognized by peers. This also seems to have influenced the client-clinician relationship, as young adults not only felt recognized by their clinicians but also were more willing to engage. Given the challenges of working with a young street-involved population, THN can serve as an important engagement tool.

Study participants had several recommendations, in both policy and practice, for quality improvement. One specific benefit of using a CBPR design is the ease and speed with which participant recommendations can effect change and be quickly put into practice [24]. Specific recommendations from the young adult participants included improving the training video, using simulation dummies and offering repeat/refresher training. Some of these changes have been implemented both locally and provincially. For instance, the recommendation to increase access to THN training led ICY to establish daily trainings led by peers. Participants recommended providing and advertising THN refresher courses, making THN opt-out rather than opt-in for ICY clients, and continuing to encourage participation in THN for youth, young adults, and support people. In response, ICY now routinely offers refresher courses when a young person requests a replacement kit, expresses interest in repeating the training, or describes risky behaviors amongst themselves or their peer group. At the provincial level, a new youth-specific video has been developed with input from the young adult peer researchers and participants [25].

Another important aspect of CBPR is the sharing of research findings in an accessible manner back to the community. A graphic booklet using direct quotes from this study illustrated by ICY participants was created and disseminated amongst youth, young adults, families, and service providers. Postcards using the same illustrations and quotes from the booklet and including information on how to recognize and respond to an overdose on the reverse side have also been developed and distributed [26].

Limitations of this study include a small sample size and exclusion of youth under the age of 19. However, participants included males and females and reported varied duration of opioid use and a variety of other substances used. The dual roles of some research team members as researchers and staff clinicians in this study presented an ethical challenge of recruiting eligible young people while protecting their identity from researchers involved in their clinical care.

Trustworthiness of the findings is enhanced by including experiential peers in the research and analysis processes. The peer researchers confirmed interpretation of the themes and helped to create relevant and acceptable knowledge disseminations tools. This study suggests that mental health and addiction programs that provide overdose response training empower street-involved young adults to care for their health and their communities. Programs that communicate respect and capacity help young people understand that they are valued, even if they continue to engage in risky behaviors. This approach to engagement could improve other health and harm reduction programs for this population.

## Conclusions

Previous findings of THN programs have shown that it is an effective harm reduction intervention. The findings from this qualitative study support additional benefits of THN programs in a street-involved young adult population as findings suggest improved safety, altruism, and empowerment, and improved self-esteem because of training. Other benefits include increased young adult engagement with health care staff that provide THN, and with other aspects of health care programming. The CBPR process also generated participant recommendations, which have been incorporated into the provincial and local THN initiatives.

## Additional files

Additional file 1: Focus Group Questionnaire. (DOC 34 kb)

Additional file 2: Individual Interview Questionnaire. (DOC 28 kb)

## Abbreviations

$ CDN: Canadian dollars
BC: British Columbia
BCCDC: British Columbia Centre for Disease Control
CBPR: Community-based participatory research
CNS: Central nervous system
DSM-5: Diagnostic and statistical manual, fifth edition
ICY: Inner City youth program
MPH: Masters of public health
SRO: Single room occupancy
THN: Take home naloxone
