# Supplementary material for: Naloxone and the Inner City Youth Experience (NICYE): a community-based participatory research study examining young people’s perceptions of the BC take home naloxone program
Source: Harm Reduct J. 2017 Jun 7;14:34. doi: 10.1186/s12954-017-0160-3 (PMC5463299; doi:10.1186/s12954-017-0160-3)
Supplement: Supplementary file 1 — Focus Group Questionnaire. (DOC 34 kb) [file 12954_2017_160_MOESM1_ESM.doc]

**Additional file 1 – Focus Group Questionnaire**

# Project Title:

# Naloxone and the Inner City Youth Experience “NICYE”

**NICYE Focus Group and Interview Guide**

In this focus group, we are going to talk about your experience with the Take Home Naloxone (THN) program.

**(Training)**

First, we are going to ask you about the THN training that was offered to you by the Inner City Youth (ICY) program. Remember, if you feel uncomfortable at any point, you can pause or end the interview.

**1. How did you find out about the Take Home Naloxone training?**

- How did it get brought up?
- What was that like for you?

**2. When did you do the THN training?**

- When you say … (ie. long time ago, etc), what do you mean by that?

**3. Why did you take the THN training?**

**4. Can you tell me what the THN training was like?**

- What happened during the THN training?
- What happened then/next?
- How was the THN training for you?
- Do you remember who did the THN training with you? (ie. Case manager or Nurse Practitioner)
- What happened at the end of the THN training?
- What happened after you left the THN training?

**5. What can make the THN training better?**

- Can you tell me more about how ‘x’ would make it better?

**6. Did you tell anyone about the THN training?**

- Who did you tell?
- How did they respond?

**7. After the THN training, how prepared did you feel to respond to an overdose?**

**Overdose**

At this point, we are finished talking about the THN training offered by ICY. Now, we are going to switch the discussion towards overdose. Given that this can be a sensitive topic for many people, we just want to clarify that you can pause or end the interview if you are feeling uncomfortable. Also, we want to remind you that ICY support staff will be available for anyone needing support after the interview.

**8. What kind of experience have you had around overdose?**

This refers to your own experience or that of others

**9. Can you tell me about your thoughts or feelings after the experience?**

**10. What was it like using the kit?**

- When you say … (ie. Difficult, easy, etc) what do you mean by that?

**11. What happened after you used the kit?**

- What happened when you wanted to get the kit refilled?

**ICY Engagement**

At this point in the questions, we are shifting our focus from discussing overdose to the ICY program.

**12. What does it mean to you that ICY provides THN training?**

**13. What was your interaction or experience with ICY like after the training?**

**14. What happened with the way you talk about your drug use or mental health with your ICY worker(s) since having taken the THN training?**

Thank you for all of your responses. At this point in the interview, we are finished with our questions. Do you have anything else you want to add to what we have talked about?

Thank you for taking part in the focus group. We appreciate you taking the time to talk with us. Again, if any of you feel that you would like to follow up with an ICY support worker because of some of the sensitive topics brought up, we are able to direct you to the appropriate support staff. Thanks again.
