# Supplementary material for: Naloxone and the Inner City Youth Experience (NICYE): a community-based participatory research study examining young people’s perceptions of the BC take home naloxone program
Source: Harm Reduct J. 2017 Jun 7;14:34. doi: 10.1186/s12954-017-0160-3 (PMC5463299; doi:10.1186/s12954-017-0160-3)
Supplement: Supplementary file 2 — Individual Interview Questionnaire. (DOC 28 kb) [file 12954_2017_160_MOESM2_ESM.doc]

**Additional file 2 – Individual Interview Questionnaire**

# Project Title:

# Naloxone and the Inner City Youth Experience “NICYE”

**Interview Question Guide**

1. When were you trained in overdose prevention and Take Home Naloxone?
2. What was it like to be given a certificate that recognized you know how to respond to an overdose?
3. Did you have any support people trained also? If so, then who?
4. Do you still have your kit?
   1. Where do you keep your kit?
5. How many kits have you been given?
6. Have you ever witnessed an overdose?
   1. If yes, Have you witnessed an overdose since receiving the training?
   2. Have you ever responded to an overdose, including by giving naloxone?
7. How confident do you feel in your ability to respond to an overdose?
   1. 1 - not at all confident 5 – neutral 10 – very confident
   2. What would improve your confidence?
8. How confident are you that naloxone would work for an opioid overdose?
9. What was it like to receive this overdose prevention training?
   1. What did you like about the training? What was important?
   2. What did you learn that was helpful about overdose prevention and harm reduction?
   3. Is there anything that would have improved the training?
   4. Is there anything you didn’t like about the training? Ways it could be improved?
10. Did this overdose prevention training change anything about the way you use drugs?
    1. If yes, then in what ways have you changed your drug use?
11. Has having naloxone available changed the amounts of drugs you use?
    1. If yes, how has it changed?
12. How did this training affect your feelings about the ICY team?
    1. Did it affect how interested you were in continuing to connect with ICY staff to discuss your health?
13. Is this something you would recommend to other youth?
    1. Why or why not?
14. Is there anything else you think is important for us to know?
